# Development of a Prototype Lateral Flow Immunoassay of Cortisol in Saliva for Daily Monitoring of Stress

**DOI:** 10.3390/bios11050146

**Published:** 2021-05-07

**Authors:** Elizaveta Panfilova

**Affiliations:** Institute of Biochemistry and Physiology of Plants and Microorganisms, Russian Academy of Sciences, 410049 Saratov, Russia; panfilova_e@ibppm.ru; Tel.: +7-8452-97-0403

**Keywords:** immunochromatographic test, emotional stress, salivatory cortisol, point-of-care testing, rapid detection, gold nanoparticles conjugation

## Abstract

Emotional stress negatively affects the quality of a person’s daily life. From a physiological point of view, stress is expressed in the excitation of the hypothalamic–pituitary–adrenal cortex axis, which leads to the release of the hormone cortisol into the blood. We developed a lateral flow immunoassay to detect cortisol in human salivary fluid and tested it on 10 healthy volunteers daily for about one month (*n* = 293 saliva samples). Cortisol was detected in concentrations ranging from 1 to 70 ng/mL. Salivary cortisol levels were confirmed by ELISA. The straightness range of LFIA calibration was from 1 to 100 ng/mL. The diagnostic sensitivity of the method was 73%. It was found that in 3 out of 10 subjects, fluctuations in the level of cortisol in saliva partially corresponded to the subjectively assessed level of stress.

## 1. Introduction

Lateral flow immunoassays are firmly established in daily analytical practices due to their advantages such as fast results, simplicity in use, and the wide range of substances they can detect. At the moment, immunochromatographic tests have already been introduced to determine pregnancies, detect sexually transmitted diseases, identify drugs in urine, diagnose COVID-19, intestinal infections, influenza, rotavirus, etc. Researchers are developing immunochromatographic tests to identify pathogens [1,2] and markers of various diseases in humans [3,4,5,6], animals [7], and plants [8], pesticides [9], and toxins in water and food [10]. Immunochromatographic tests are also being developed to detect human hormones in order to assess health statuses [11,12,13].

Cortisol, a corticosteroid hormone produced by the adrenal glands, is interesting in that its level rises as a result of the body’s response to stress. Situations that a person constantly encounters lead to an increase in cortisol levels and include medical intervention [14,15], disturbance of circadian rhythms [16], physical impact, such as noise [17], and emotional stress [18,19,20,21,22,23,24,25]. Emotional stress can be triggered by the events of the day that involve the emotional ego and/or anxious anticipation of a negative event [20], such as the need to speak to an audience [18] or the anticipation of a painful surgery [19].

Various sources have consistent data on an increase in the concentration of cortisol in morning saliva due to a stressful state of the body. Thus, in [25] the authors show that in a fairly large sample (more than 300 people in each group), study participants with anxiety disorders show higher levels of cortisol than participants in the control group without anxiety disorders. Studying a group of men between the ages of 27 and 57 from the so-called “white collar” workers, the authors also found that anxiety and depression are associated with a significant level of elevated cortisol; in addition, they also demonstrated that a negative effect on mood, emotional arousal, and the stressful events of the day are also accompanied by increased cortisol levels [26].

Detection of cortisol in saliva seems to be convenient due to non-invasiveness and minimization of interference in the personal space of the subject. Currently, in clinical practice, ELISA is used to determine cortisol in saliva. ELISA is a rather complex method, since it requires trained personnel, numerous reagents, compliance with the storage conditions for reagents, equipment, data analysis, and statistical analysis. Researchers in recent years have proposed various methods, including a lateral flow immunoassay [27,28], bioluminescent probes [29], a portable surface plasmon resonance (SPR) biosensor system [30], a time-resolved fluoroimmunoassay [31], a luminescence immunoassay [32], and colorimetric analysis [33] for the rapid detection of cortisol in human biological fluids, including saliva.

Despite the presence of publications devoted to the creation of a test for detecting cortisol using immunochromatographic analysis of human biological fluids, including saliva [27,34], sweat [35], and blood plasma [28], so far there have been no publications containing both the development of the method and analysis of a representative sample, which is necessary to demonstrate the effectiveness of the application of the method in practice. In this work, we developed an immunochromatographic test, starting from obtaining a conjugate of GNPs with antibodies to cortisol, selecting the optimal parameters for constructing a test system, and ending with the analysis of 287 saliva samples obtained from 10 volunteers during a period of about a month. The aim of this work was to obtain a profile of changes in cortisol levels over a month and compare it with subjectively assessed stress.

## 2. Materials and Methods

### 2.1. Reagents and Materials

Tetrachloroauric acid (HAuCl_4_, 99.99%) was purchased from Alfa Aesar (Haverhill, MA, USA). Bovine serum albumin (BSA) was obtained from Amresco (Solon, OH, USA). Mouse anti-cortisol antibodies, cortisol–BSA, and goat anti-mouse antibodies were purchased from Bialexa (Moscow, Russia). Human serum albumin (HSA) was purchased from Reanal (Hungary), and human immunoglobulin was obtained from Biotechnologies Innovation Center (Russia). Tween 20 and sodium citrate were purchased from Sigma–Aldrich (St. Louis, MO, USA). All reagents were of analytical or chemical purity. All chemicals were used without further purification. A synthesis of gold colloid and its conjugation with antibodies was prepared in deionized water (simplicity system, Millipore; Bedford, MA, USA; specific resistivity at 25 °C was ≥18.2 MΩ cm). For lateral flow test strips, the following membranes were used: a nitrocellulose (NC) membrane grade CNPF with a pore size of 8 µm attached to a solid support, conjugate release matrix PT-R7, a sample membrane GFB-R7, and an absorption membrane AP 045 (all membranes from Advanced Microdevices; Ambala Cantonment, India). ELISA was performed using a Cortisol Saliva ELISA kit from DBC (London, ON, Canada) and a spectrophotometer Specord S300.

### 2.2. Synthesis of Gold Nanospheres and Conjugation with Antibodies

**Figure A1:**
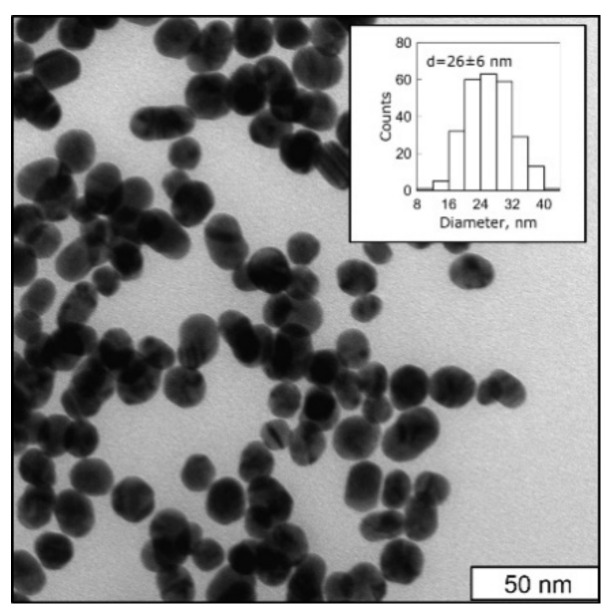
TEM image and size distribution of synthesized Au nanoparticles.

All glassware used was cleaned with 3:1 HCl:HNO_3_ and was rinsed thoroughly in H_2_O before use. The synthesis was prepared in a 100 mL Erlenmeyer flask with a reflux condenser. For preparation of 25 nm Au nanospheres, we used the standard protocol [36]. As such, 3.5 mL of 1% sodium citrate was added to 244 mL of boiling deionized water. Then the stirrer speed was increased, and 2.5 mL of a 1% solution of hydrochloric acid was added. The reaction mixture was boiled for 15 min. As a result, we obtained a colloid of gold nanospheres with a size of 26 ± 6 nm. The TEM image of the resulting nanoparticles and a histogram of size distribution are shown in Figure A1 (Appendix A).

The fabricated Au nanospheres were characterized by absorption spectroscopy with a Specord S300 spectrophotometer and by transmission electron microscopy (TEM) with a Libra 120 instrument (Carl Zeiss, Jena, Germany).

To prepare the conjugate, the pH of the Au colloid was adjusted to 8 using 0.2 M K_2_CO_3_. After that, 50 μL of monoclonal antibodies (Ab) to cortisol (Bialexa, Russia) were added to 500 μL of Au colloid. The final concentration of Ab was 30 μg/mL. After incubation for one hour, 50 μL of 1% bovine serum albumin (BSA) was added, and after 10 min, the conjugate was centrifuged at 4 °C 8000 rpm for 15 min. Conjugate was redispersed in 10 mM of a phosphate salt-free buffer. O.D. of the prepared conjugate colloid was 1 ± 0.05.

### 2.3. Saliva Samples Preparation: Participant Characteristics

Unstimulated saliva samples were collected in the morning immediately after waking up, before eating and drinking, and before smoking. Immediately before collecting the saliva, each study participant rinsed their mouth with water. The saliva was taken into a sterile beaker for biomaterial sampling. The saliva was stored until analysis in a freezer at −20 °C.

The study included 10 adults: 4 men and 6 women aged 25 to 39 years. None of the participants were smokers. All participants were Russian. The average age was 31.4 ± 3.7. Of the participants, 9 out of 10 were married, and 4 of 10 participants had children. Furthermore, 8 of 10 participants were of normal or underweight and 2 were overweight. Participant no. 3 was taking medication to normalize their digestive system.

Each participant was asked to rate their subjective level of yesterday’s stress. The responses were scored similarly to in [37,38] on a 5-point scale from 0 = no stress to 5 = very high stress. During the study, participants noted their stress levels every day.

### 2.4. Preparation of Lateral Flow Immunoassay Test Strip and Assay Performing

Before application, the membrane conjugate pad was immersed into a solution containing 0.01% Tween 20 and 0.01% PVA (9–10 kDa) and dried in a thermostat at 35 °C. For detailed instructions on how to assemble the test strips, see article [28]. After the assembling of the pads onto the membrane, the membrane was cut into 5 mm strips using a paper guillotine. A half μL of the cortisol–BSA complex (1.05 mg/mL) was applied onto the membrane as the control zone and 0.5 μL of anti-species antibodies (200 μg/mL) was applied as the test zone. The test strips were dried in the thermostat at 35 °C for 3 h.

Before the assay was performed, saliva samples were centrifuged at 2000 rpm for 15 min. After that, 10 μL of the colloidal gold conjugate was added to 60 μL of the sample and incubated for 1 h, before 70 μL of the mixture was applied to the sample pad. Although the conjugate was not applied separately to the conjugate pad, this pad was still used because its use reduces the background. The analysis took place over 15 min, and after this time the intensity of staining of the spots did not change. All test strips were dried at room temperature and scanned using the Epson Perfection V700 Photo (Seiko Epson Corp., Jakarta, Indonesia).

### 2.5. Plotting a Calibration Graph: Calculation of Results

The containment of cortisol in cortisol–BSA complex was determined as 18.6% [39]. A calibration graph was plotted using 10 solutions of cortisol–BSA with concentrations of cortisol from 0.001 ng/mL to 10 mg/mL. All obtained data were calculated using Image J. The mean intensity of the higher spot was determined as the mean intensity of each pixel and used as the intensity of the test zone (T). The mean intensity of the lower spot was determined as the mean intensity of each pixel and used as the intensity of the control zone (C). All measurements were conducted in triplicate. The ratio T/C was plotted versus the cortisol concentration C_cort_ to make a calibration graph.

## 3. Results and Discussion

**Figure 1:**
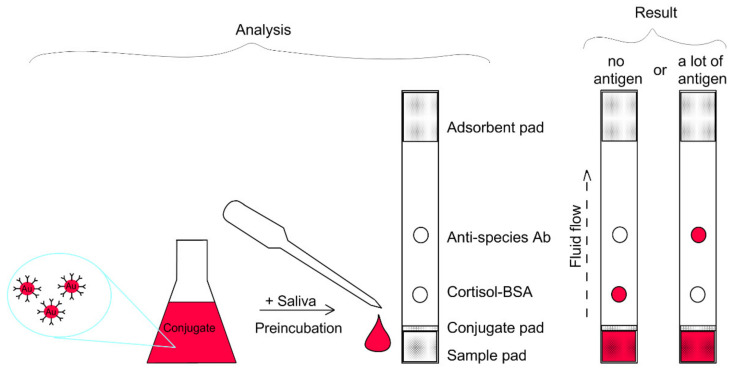
Scheme of immunochromatographic analysis in a competitive format. On the (**left**), the analysis procedure is shown, which includes preincubation of the sample with the conjugate, as well as the scheme for applying the reagents to the test strip. On the (**right**), two extreme cases for the analysis result are shown: the absence of antigens in the sample, and a large quantity of antigens.

The immunochromatographic analysis is based on the specific antigen–antibody interaction. In order for this interaction to be detected with the naked eye or with a device, the antibody molecules are “labeled” with a definite type of marker, in our case, colloidal gold. Antibody molecules attach to the surface of the gold nanoparticle through physical adsorption. Such “labeled antibodies” are a conjugate, which, like colloidal gold, is a colloid. This colloid is incubated with a specified amount of the sample containing the analyte. After a certain period of time, the resulting mixture of the conjugate and the sample is applied to the sample pad of the test strip, then by means of capillary forces, the mixture moves up the test strip, simultaneously interacting or not interacting with the molecules applied to the membrane of the test strip. The fact of interaction is recorded as a staining of the spot on the test strip in red (colloidal gold color). To determine low molecular weight substances, a competitive method is used. In this method the lower spot is the applied complex of this substance (the antigen) with the carrier protein, and the upper spot is anti-species antibodies. If the sample contains a large amount of antigen, this antigen interacts with all antibody molecules in the conjugate, therefore, as the liquid passes through the test strip, the conjugate does not interact with the antigen–protein–carrier complex applied to the control zone of the strip, but enters into a reaction with the anti-species antibodies and we see staining of the upper spot. In the opposite case, if the sample contains a small amount of the antigens, the antibodies in the conjugate will react with the antigen–carrier–protein complex applied to the membrane and we will see either the staining of the lower spot, but not the upper one because the entire label “remained” on the lower spot, or a decrease/increase in the intensity of staining of each of the spots, depending on the concentration of antigens in the sample (Figure 1).

One of the difficulties of modern methods of diagnosing stress is the difficulty in capturing its chronic level [40]. A reliable express method that allows us to determine cortisol every day, at any moment, for a long period of time, can serve as a basis for understanding the formation of stress-dependent diseases in the future. We decided to use the developed test strips to monitor cortisol levels on a daily basis, and to determine if the increase in cortisol levels was consistent with the emotional stress the study participant was experiencing. To do this, we asked 10 volunteers to collect saliva samples for approximately one month.

### 3.1. Obtaining Quantitative Results of Immunochromatographic Analysis of Saliva Samples

To obtain quantitative results of the immunochromatographic analysis, we used the calibration graph method. We prepared 10 cortisol solutions with a concentration of 0.001, 0.01, 0.1, 1, 10, 31.62, 100, 1000, and 10,000 ng/mL. In our preliminary work [39], we showed that with the parameters of the immunochromatographic test used by us (the ratio of hapten:carrier protein, the distance between the test and control zones, etc.), the traditional method allows us to achieve a cortisol detection limit of 100 ng/mL, which is 1–2 orders of magnitude higher than the concentration of cortisol in saliva, and therefore, in order to shift the working range of test concentrations in accordance with the concentration of cortisol in saliva, we used the preincubation method proposed in [41]. With this method, we achieved a shift in the detection limit to 1 ng/mL cortisol.

**Figure 2:**
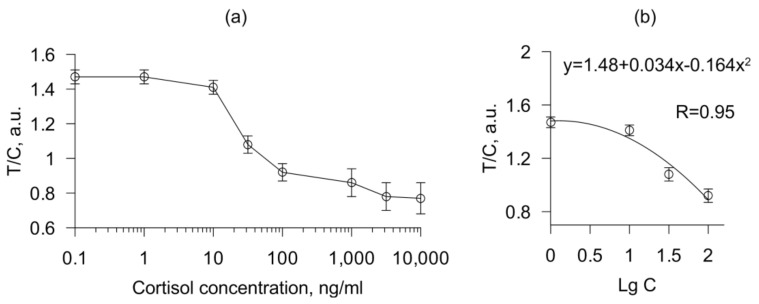
Calibration curve of the immunochromatographic analysis (**a**). A part of the calibration curve in the concentration range from 1 to 100 ng/mL was approximated by a polynomial function (**b**). The abscissa shows the concentration of cortisol (**a**) or log of the cortisol concentration (**b**) in the calibration solution and the ordinate shows the ratio of T to C. Error bars indicate the standard deviations (*n* = 3).

After applying 70 μL of the mixture of saliva+conjugate to the sample pad of the test strip, coloring was observed within 15 min. The images obtained after drying and scanning the test strips were digitized using the Image J open source software (W. Rasband, USA). The intensity of the staining of the upper and lower spots was calculated as the average of the intensity of all pixels. The data obtained were used to construct a calibration graph (Figure 2a).

We used a part of the calibration curve approximated by a polynomial function to calculate the concentration of cortisol in saliva samples. The test working range was from 1 to 100 ng/mL with a detection limit of 1 ng/mL.

**Figure 3:**
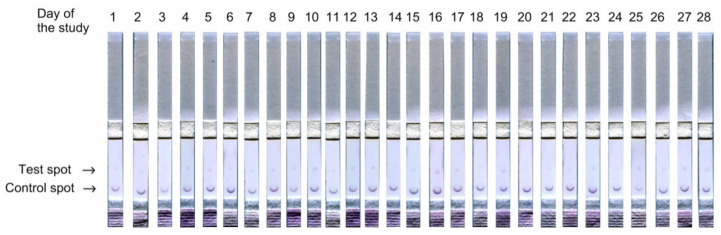
An example of test strips after examining saliva samples from study participant no. 7. It is clearly seen that the intensity of staining is different not only in the upper, but also in the lower spot.

A feature of the preincubation method is that the intensity of staining in the test zone (corresponding to the application of anti-species antibodies, the upper spot) increases with an increase in the concentration of the analyte, and the intensity of the staining of the control zone (corresponding to the application of the hapten–carrier–protein complex, the lower spot), on the contrary, decreases [42]. For example, in Figure 3, we provide images of test strips from one of the study participants, obtained by analyzing saliva samples for a month. It is clearly seen that the intensity of staining is different on different days for both the upper and lower spots.

**Figure 4:**
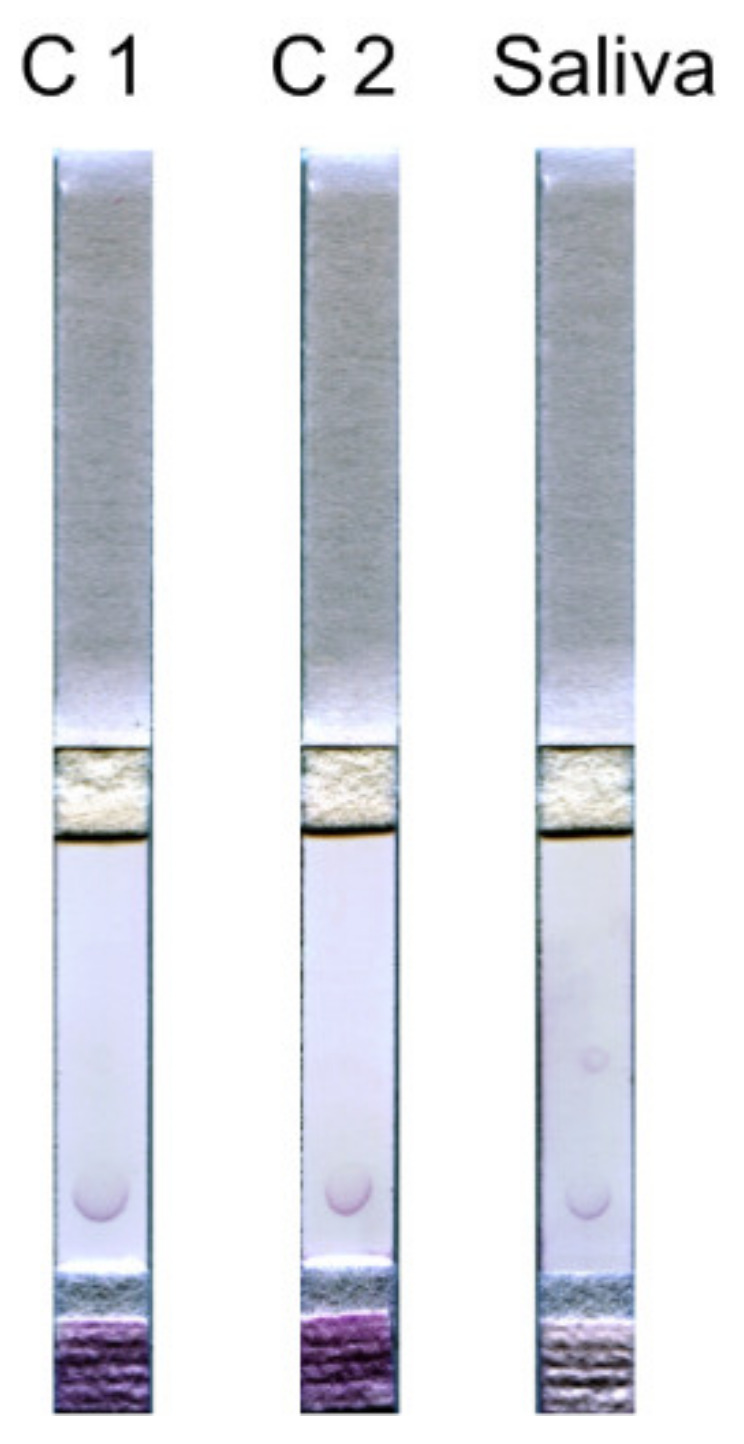
Control experiment with saliva components. C1—test performed with the HSA solution (3 mg/mL), and C2—test performed with the human immunoglobulin solution (1 mg/mL). For comparison, on the right there is a test carried out with saliva as the analyzed liquid. The test was carried out simultaneously with the control tests.

To find out if there is a cross-effect that could affect the measurement from molecules present in saliva that can provoke complexes or nonspecific reactions during the test, we performed tests with HSA and human immunoglobulin. The concentration of HSA was taken as 3 mg/mL, which corresponds to the concentration of total protein in the saliva of an adult, and the concentration of immunoglobulin was taken as 1 mg/mL, which corresponds to its physiological content in saliva [43]. The results are shown in Figure 4.

As shown by the control experiment, components of saliva such as HSA and immunoglobulin make an insignificant contribution to staining the spot, approximately at the background level.

**Figure 5:**
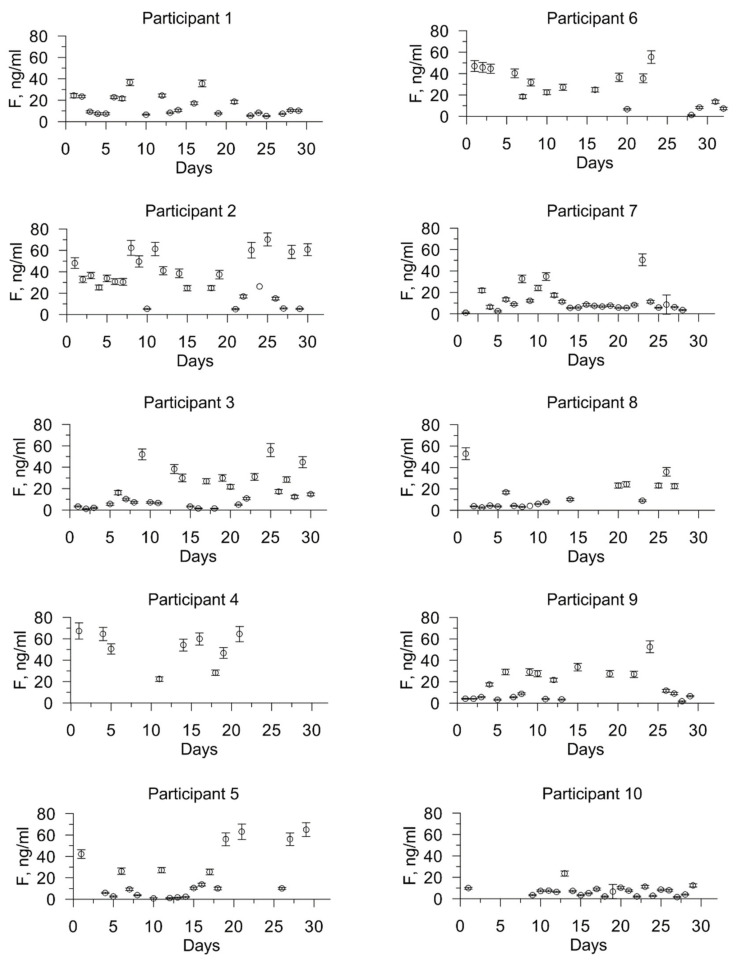
Profiles of changes in the concentration of cortisol in saliva sampled in the morning immediately after waking up for a month for each study participant.

After obtaining the calibration curve, we analyzed the saliva samples using the test strips we developed. We analyzed 293 samples from all participants. Of these, 79 samples were false negative, that is, the intensity of the staining of the upper spot corresponded by calibration to an extremely low concentration of cortisol, while the intensity of the staining of the lower spot corresponded to a higher concentration of cortisol. Thus, the diagnostic sensitivity was 73%. From the obtained values of the brightness of the spots and the calibration graph, we obtained the values of the concentration of cortisol (F) in saliva for each sample of saliva, and obtained cortisol profiles (Figure 5) over a month for each study participant.

We see that the distribution of cortisol over time is individual. The study participants are characterized by a different pattern of distribution of the level of cortisol in saliva over time: from insignificantly in comparison to other participants (from 1 to 23 ng/mL (participant no. 10)) to significant fluctuations from 5 to 70 ng/mL (participant no. 2). The mean values, as well as the maximum and minimum values, for the study period are presented for each participant in Table 1.

Regarding the maximum and minimum values of the concentration of cortisol in saliva, the following takes place. For 9 out of 10 participants, a decrease in cortisol levels to 2.2 ± 1.7 ng/mL is characteristic, but for participant number 4, not only does the cortisol level not decrease to an average minimum, but it even exceeds the average value for all participants over the study period (21.7 ± 12.9 ng/mL). This is likely to indicate chronic stress.

The method described by us includes scanning, digitizing images, and comparing the control and test spots, which seems to be quite laborious in this format. However, at present, researchers are developing smartphone readout systems for performing quantitative immunochromatographic analysis [44,45,46], the introduction of which into practice, we believe, will significantly simplify the procedure for obtaining quantitative LFIA results.

### 3.2. Comparing Emotional Stress Level and Cortisol Concentration in Saliva Samples

**Figure 6:**
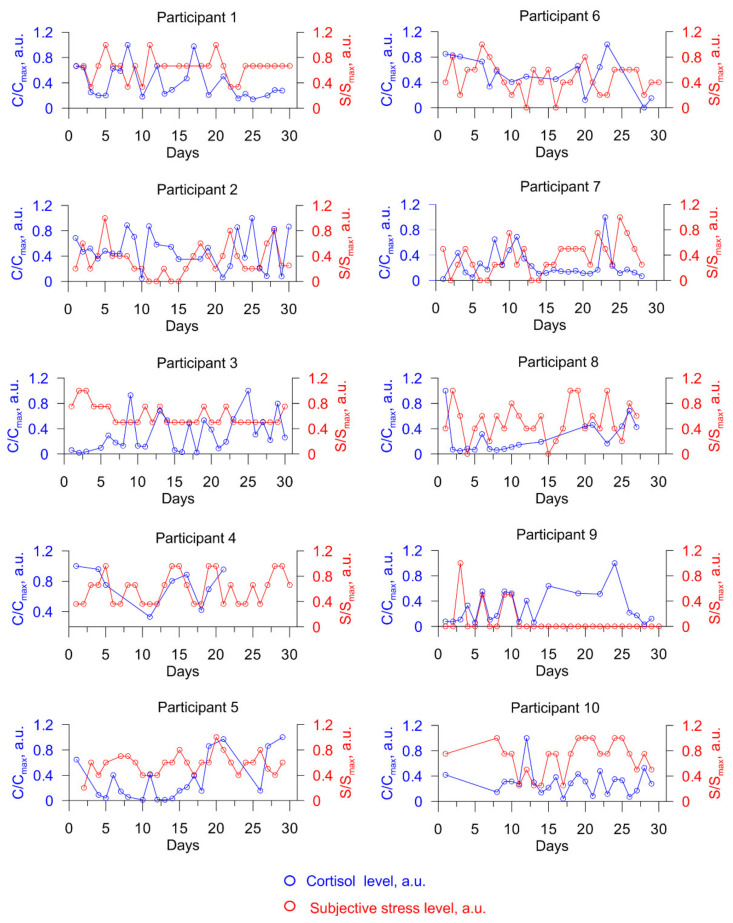
Profiles of changes in stress of each of the participants during the month. Blue denotes “cortisol” stress values expressed in C/C_max_, where C is the current value of the concentration of cortisol for each study participant, ng/mL; C_max_ is the maximum value of the concentration of cortisol in saliva for the study period for each participant, ng/mL. Red denotes emotional stress valuesexpressed in S/S_max_, where S is the current value of the emotional stress for each study participant, a.u.; S_max_ is the maximum value of the emotional stress for the study period for each participant, a.u.

Next, we decided to compare the obtained profiles of the level of cortisol with the level of subjectively perceived emotional stress. Emotional stress was rated every day during the study by the participants subjectively on a scale from 0 to 5, in accordance with their feelings. To compare subjectively experienced stress levels and cortisol levels, we divided each value of emotional stress by the maximum for each participant for the entire study period; similarly, dividing each value of the cortisol concentration by the maximum, we obtained the S/Smax and C/Cmax values, respectively. Figure 6 depicts the stress change profiles (both emotional and hormonal stress) for each study participant over the course of a month.

We can see that the profile of the change in “cortisol” stress does not exactly repeat the profile of the change in emotional stress, but several participants have separate sections of the profiles where there is a correlation. As mentioned in the introduction, increased levels of cortisol in the human body can result not only from emotional stress, but also from physical activity and environmental factors (the study participants were not limited in physical activity or isolated from the physical effects of the environment, but led a normal, daily lifestyle).

We calculated the correlation coefficient of the stress profiles for each participant without taking into account the shift in the “cortisol” profile relative to the emotional stress profile and obtained the following results. For two out of ten participants, there is a positive correlation throughout the study period (R = 0.17 (participant no. 4) and R = 0.11 (participant no. 5)). Several participants also have distinct periods with significant correlations in the stress profile, for example, participant no. 4 from days 11 to 20 of the study (R = 0.77), participant no. 9 from days 5 to 12 of the study (R = 0.87), and participant no. 5 from days 9 to 25 of the study (R = 0.26).

### 3.3. Checking Test Results Using ELISA

**Figure 7:**
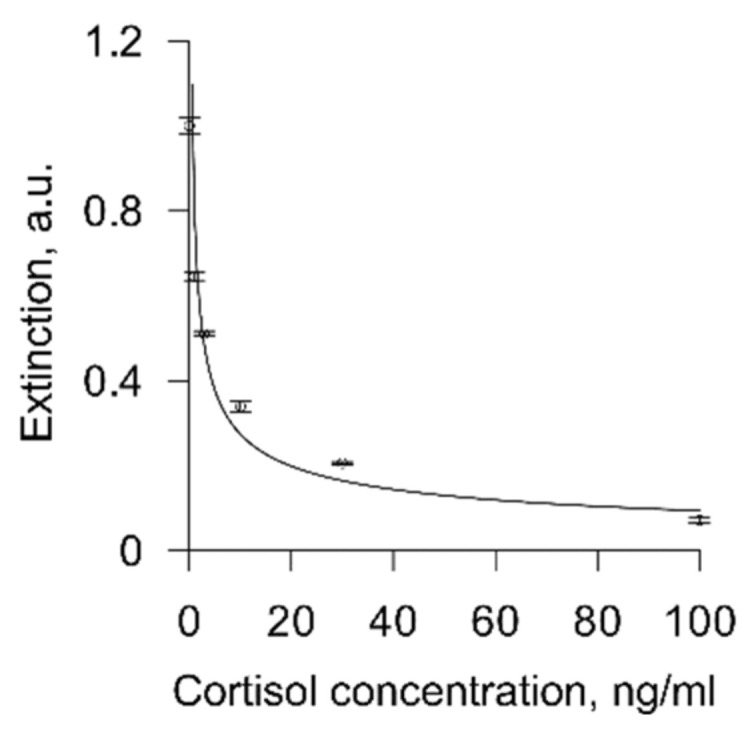
Calibration graph of the enzyme immunoassay (*n* = 3). Curve fitting equation: ln(Y) = −0.4637290342 × ln(X) − 0.2250345633. Correlation coefficient R = 0.93.

To check the correctness of the results obtained, we performed an ELISA analysis of several saliva samples. We performed LFIA and ELISA immediately to avoid errors associated with freezing or thawing of saliva, as well as possible sorption of analyte molecules by the walls of containers, etc. The ELISA was performed according to the instructions of the commercial Cortisol Saliva ELISA kit. The ELISA calibration graph is shown in Figure 7.

Next, we compared the cortisol concentrations obtained from 14 saliva samples using ELISA and the test strips we designed. The data obtained are presented in Table 2.

## 4. Conclusions

The work involved the creation of immunochromatographic strips for the determination of cortisol in saliva and their approbation with the participation of 10 volunteers for a month. The analytical sensitivity of the developed test strips was 73%. The obtained data were used to construct the profiles of fluctuations in the values of the cortisol level and the subjectively assessed level of stress. In 3 out of 10 study participants, the profiles of changes in subjective stress and changes in cortisol concentration, expressed in arbitrary units, are partially consistent. The analysis of the results obtained allows us to conclude that the test strips developed by us for the determination of cortisol may well serve as an auxiliary tool for analyzing susceptibility to everyday stressful situations and studying the effect of stress on the human body.

## Figures and Tables

**Table 1:** Average salivary cortisol concentrations for each study participant over the study period.

Participant Number	Average Cortisol Concentration During the Study Period ± SD, ng/mL	The Min/Max Value of Cortisol During the Study Period, ng/mL
1	14.9 ± 9.6	5.2/36.6
2	34.9 ± 19.6	5.1/70.3
3	17.9 ± 16.1	1.0/55.0
4	50.9 ± 16.1	22.3/67.3
5	21.6 ± 22.5	1.0/65
6	27.5 ± 16.4	1.1/55.4
7	12.3 ± 11.4	1.0/50.4
8	14.3 ± 13.8	2.6/52.9
9	15.8 ± 13.8	1.5/52.6
10	7.2 ± 4.7	1.6/23.5

**Table 2:** Salivary cortisol content determined using developed LFIA test strips and ELISA.

Sample No	LFIA ± SD, (ng/mL) Mean (*n* = 3)	ELISA ± SD, (ng/mL) Mean (*n* = 3)
1	15.9 ± 1.2	11.4 ± 0.2
2	12.3 ± 0.9	8.3 ± 0.1
3	23.5 ± 0.7	23.5 ± 1.1
4	22.5 ± 0.9	22.6 ± 0.3
5	18.6 ± 0.5	21.8 ± 1.1
6	17.2 ± 0.1	16.0 ± 1.5
7	5.8 ± 0.4	7.3 ± 1.8
8	27.7 ± 0.7	24.3 ± 1.1
9	38.3 ± 2.4	35.8 ± 3.2
10	24.0 ± 1.4	23.0 ± 0.3
11	28.2 ± 2.1	25.0 ± 0.8
12	35.4 ± 1.3	32.5 ± 2.5
13	25.0 ± 1.6	21.2 ± 0.9
14	60.9 ± 3.1	63.1 ± 3.0

The correlation coefficient was 0.98, which indicates good agreement between the two methods.
